# Spontaneous Heterotopic Triplet Pregnancy with a Two Viable Intrauterine Embryos and an Ectopic One with Right Tubal Rupture

**DOI:** 10.1055/s-0039-1683910

**Published:** 2019-04-10

**Authors:** Adriano Carvalho Guimarães, Luciano Dias de Oliveira Reis, Fabio Chaves Leite, Cassiana Franco Dias dos Reis, Alex Paula Costa, Walter Junior Boim de Araujo

**Affiliations:** 1 V&P Health Excelência Médica, Santo Antônio da Platina, PR, Brazil; 2 Hospital Nossa Senhora da Saúde, Santo Antônio da Platina, PR, Brazil; 3 Hospital Regional do Norte Pioneiro, Santo Antônio da Platina, PR, Brazil; 4 Instituto da Circulação, Curitiba, PR, Brazil

**Keywords:** obstetrical surgery, pregnancy complications, heterotopic pregnancy, multiple pregnancy, tubal pregnancy, cirurgia obstétrica, complicações na gravidez, gravidez heterotópica, gravidez múltipla, gravidez tubária

## Abstract

Heterotopic pregnancy (HP) is defined as the simultaneous development of an intra- and an extra uterine gestation. The occurrence of a spontaneous triplet HP is an exceptionally rare medical condition. We report the case of a young woman with spontaneous heterotopic triplets at 8 weeks of gestation, with a misdiagnosis of topic twins and acute appendicitis. The ectopic tubal pregnancy was ruptured and a salpingectomy was performed by laparotomy. The intrauterine pregnancy progressed uneventfully. The two healthy babies were delivery by cesarean section at 36 ± 2 weeks of gestation. Heterotopic triplets with ruptured tubal ectopic pregnancy represent a special diagnostic and therapeutic challenge for the obstetrician. A high rate of clinical suspicion and timely treatment by laparotomy or laparoscopy can preserve the intrauterine gestation with a successful outcome of the pregnancy.

## Resumo

A gravidez heterotópica é definida como o desenvolvimento simultâneo de uma gestação intra- e extra-uterina. A ocorrência de gravidez tripla heterotópica espontânea é uma condição médica excepcionalmente rara. Relatamos o caso de uma jovem com gravidez tripla espontânea, às 8 semanas de gestação, com um diagnóstico errôneo de gêmeos tópicos e apendicite aguda. A gravidez tubária ectópica estava rota e uma salpingectomia foi realizada por laparotomia. A gravidez intrauterina progrediu sem intercorrências. Os bebês nasceram saudáveis por cesariana realizada às 36 semanas de gestação. A gravidez de heterotópicos com ectopia e rotura tubária é um desafio diagnóstico e terapêutico. Um alto índice de suspeita e tratamento oportuno por laparotomia ou laparoscopia podem preservar a gestação intrauterina com um resultado bem sucedido da gravidez tópica.

## Introduction

Heterotopic pregnancy (HP) is a rare medical condition in obstetrics. It is characterized by the presence of coexistent intrauterine and ectopic pregnancies. The most frequent implantation site of the ectopic pregnancy is the fallopian tube, most commonly in its ampullary segment (80%).^1^ ^2^ The incidence of HP is around 1:30,000, in spontaneous pregnancies.^1^ ^2^ ^3^ In pregnancies resulting from assisted reproduction techniques (ART), the incidence is greater, ranging from 1:100 to 1:3,600, nearly as high as 1% in some series.^1^ ^2^ Overall, the incidence of HP nowadays is estimated around 1:7,000^2^ to 1:15,000 live births (0/8% calculated risk) in contrast with the lower incidence of 1:30,000 in 1948.^4^ The higher incidence of HP in patients under ARTs programs is attributed to multiple ovulation, higher incidence of pelvic inflammatory disease (PID) observed currently, and tubal damage related or not to transfer of many embryos.^1^ ^5^ The twin rate increased from 1.8% in 1971–77 to 2.8% in 1998, attributable to ART extended use.^6^ Heterotopic triplets are even more uncommon, and cases with tubal ectopic and coexisting twin intrauterine pregnancy are limited. This medical condition can be hazardous to the intrauterine pregnancy as well as to the life of the mother. We present the case of a ruptured right tubal pregnancy, referred as acute abdomen in a patient pregnant with intrauterine twins and suspected acute appendicitis. The final outcome was the birth of healthy twins. The aim of this paper is to emphasize the need for high clinical suspicion of this clinical entity during the routine first trimester ultrasound examination, even in the presence of an intrauterine multiple gestation and especially when predisposing factors such as *in vitro* fertilization (IVF) are present.

## Case Description

**Fig. 1:**
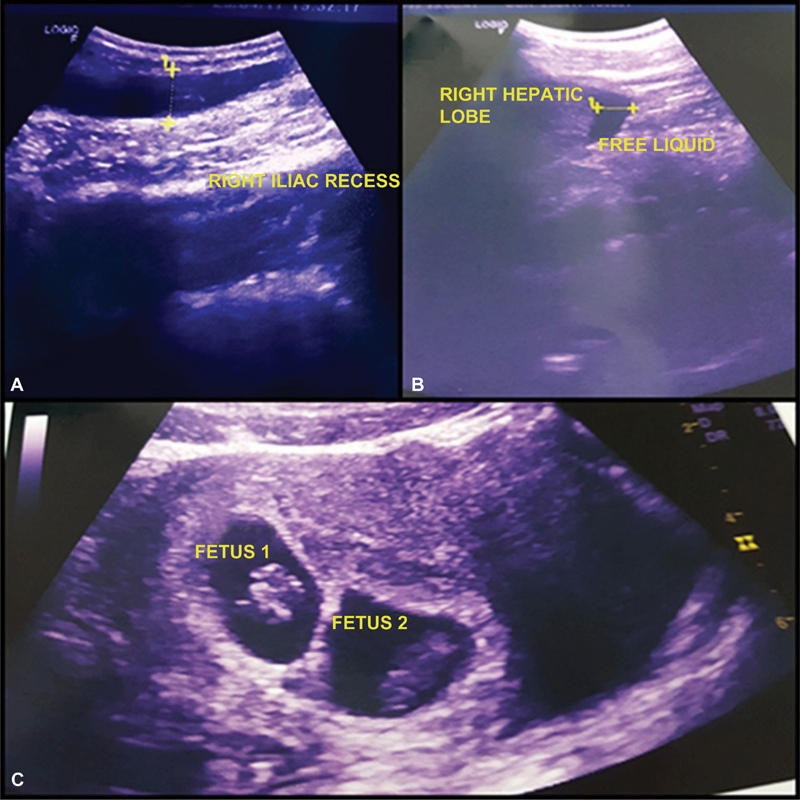
An ultrasonography had shown a 2 centimeters tubular structure in the right iliac fossa reported as acute appendicitis (A), presence of fluid in the perihepatic space (B), and 8-week viable intrauterine twin gestation (C).

A 21-year-old woman, primigravida, with a confirmed intrauterine twin pregnancy, was referred to the emergency department of our hospital by her family doctor, presenting with abdominal pain in the right iliac fossa, with diagnosis of acute appendicitis, on April 25, 2017 at 8 PM. On admission, she was stable, with a normal level of consciousness. The pulse rate of the patient was 97/min, and her blood pressure was 100/60 mmHg. The level of hemoglobin was 8.5 g/dl and leukocytosis was observed (23.700/uL). An ultrasonography had shown a 2-cm tubular structure in the right iliac fossa, reported as acute appendicitis, and an 8-week viable intrauterine twin gestation (Figs. 1A, 1B, 1C).

**Fig. 2:**
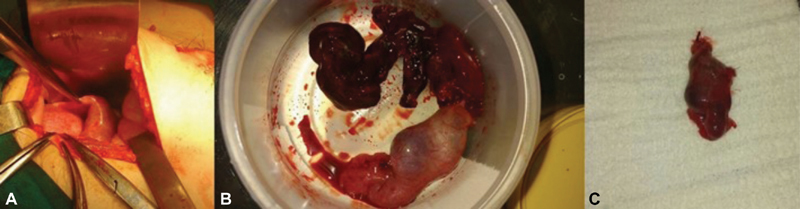
Intraoperative image of right uterine tube (A). Anatomical specimen of right uterine tube with ectopic fetus (B and C).

Physical examination demonstrated marked right iliac fossa tenderness and rebound. After examination by the surgeon on call, the patient was immediately sent to the operating room and underwent an emergency laparotomy, via Mcburney incision, during which an organized hematoma was encountered. A normal appendix was found and a midline infra umbilical second incision performed. A ruptured right ectopic pregnancy was confirmed, and a right salpingectomy was performed (Fig. 2).

The postoperative recovery was uneventful, and the patient was discharged on the third day. A transvaginal ultrasonography on the second postoperative day showed viable topic twins with crown-rump length measurements of 17 and 17.2 mm, compatible with an 8 ± 1 weeks pregnancy. Histopathological examination confirmed the diagnosis of ectopic tubal pregnancy. The obstetric follow-up and fetal assessments were normal, with normal fetal growth of each twin until the 24^th^ week. The intrauterine babies were born healthy by cesarean section at 36 weeks of gestation.

## Discussion

Heterotopic triplets are rarely encountered in everyday clinical practice. However, the extended use of ART procedures nowadays has increased the ectopic and, subsequently, the HP rates. This clinical entity represents a potentially life-threatening condition for both the mother and the intrauterine pregnancy^2^ and was first reported by Marshal in 1903.^7^ Predisposing factors to HP are identical to those predisposing to ectopic pregnancy: factors related to IVF, like large number of transferred embryos, a transfer near the uterine horn, excessive pressure on the syringe and deep insertion of the catheter during transfer, the quality of the embryos, the hormonal milieu at the moment of transfer, the use of gonadotropins, the amount of fluid used as media for the embryos, and also adhesions related or not to endometriosis and PID.^8^ ^9^ In our case, the patient had no history of PID or infertility. She was pregnant for the first time, resulting in low suspicion of HP.

The implantation of an embryo in the wall of the fallopian tube bears a high risk of rupture because the rich local vascularization and trophoblastic invasion may cause tubal rupture, even if there is no fetal cardiac activity. In cases of HP following IVF, the diagnosis can be exceptionally difficult. The Beta-human chorionic gonadotropin (β-hCG) may continue to rise normally, the ovaries may present enlarged, the ectopic gestational sac can easily be missed on ultrasound scan, and the intermittent unilateral pain can be attributed to a hemorrhagic corpus luteum or ovarian hyper stimulation.^10^ It is reported that approximately 70% of heterotopic pregnancies are diagnosed between 5 and 8 weeks of gestation, 20% are diagnosed between 9 and 10 weeks, and the remaining 10% are diagnosed after 11 weeks.^11^ Only in 57% of the cases presented in the literature the diagnosis of heterotopic triplets was preoperatively made.^11^ Around 50% of heterotopic pregnancies are asymptomatic.^3^ Most of them (78.5%) were diagnosed after the rupture of the tube, with acute abdomen symptoms.^5^ Abdominal pain due to peritoneal irritation is the most frequent symptom appearing in 82.7% of heterotopic pregnancies. Cases of heterotopic triplets with bilateral tubal pregnancy and an intrauterine pregnancy have been reported in the literature, and symptoms may, in such cases, diffuse abdominal pain.^12^ We identified a total of 11 cases of spontaneous HP, 6 of which were included in the review by Bataille et al,^13^ as shown in Table 1 below. The most common symptom was low abdominal pain, the most commonly diagnosed method was ultrasonography, and the most widely adopted surgical procedure was laparotomy with salpingectomy. Yet, of all cases evaluated, only10 resulted in the baby being born alive and healthy.

**Table 1:** Reported heterotopic triplets in the literature

Authors	Year	Age (years)	G/A (weeks)	Symptoms	Ultrasonographic signs	Tubal rupture	Treatment	Outcome
Marshal^7^	1903	30	± 15.5	Pain in the lower abdomen; swollen abdomen; pallor; vomiting; Tachycardia	———–	Yes	Hysterectomy	Mother and children have died
Selo-Ojeme and GoodFellow^4^	2002	27	7	Abdomen tenderness; Tachycardia	Intrauterine pregnancy with a crown rump length of 10 mm. The right ovary was normal. The left ovary was enlarged and contained a 33 mm cyst	Yes	Emergency laparoscopy surgery	Spontaneous vaginal delivery at 39 weeks
Pan et al.^12^	2002	38	5-7	Lower abdominal-pelvic pain, nausea, tenesmus, vaginal bleeding	Bilateral multicystic ovaries with accumulation of fluid in cul-de-sac	Yes	Salpingectomy by laparotomy	Delivery at term
Bugatto et al.^5^	2010	28	12	Abdominal pain in the left iliac region	Intrauterine two pregnancy with a crown rump length of 48 e 53 mm. The third gestational sac of 28mm could also be observed in the left uterine adnexa	No	Salpingectomy by laparotomy	Delivery by cesarean at 31 weeks
Basile et al.^1^	2012	28	7	Lower abdominal-pelvic pain	The intrauterine gestational sac according to 7 weeks of pregnancy, in addition, there was a left ovarian mass suggestive of an ectopic pregnancy	Yes	laparoscopy surgery	viable pregnancy, at 3rd trimester
Alsunaidi^14^	2005	42	8	Abdominal pain, tachycardia	Hemoperitoneum, latero-uterine sac with 2 yolk sacs	Yes	Salpingectomy by laparotomy	She delivered a healthy girl at 39 weeks
Cholkeri-Singh and LaBarge^11^	2007	30	5,5	Abdominal pain, nausea and vomiting	Hemoperitoneum, ovary not viewed	Yes	Salpingostomy by laparoscopy	Cesarean section with healthy twins at 34 weeks
Simsek et al.^15^	2008	37	9	Diffuse lower abdominal tenderness, peritoneal irritation	Hemoperitoneum, latero-uterine gestational sac	Yes	Salpingectomy by laparotomy (twin tubal pregnancy)	Delivery at term
Jeong et al.^16^	2009	34	6	Skin pallor, distended abdomen, hypotension, tachycardia	Hemoperitoneum, right hypoechoic cyst	Yes	Bilateral Salpingostomy by laparoscopy	Evacuation of intrauterine gestational sac by dilatation and curettage
Arsala and Danso^17^	2014	27	4	Tachycardia, abdominal pain, with distended abdomen	Hemoperitoneum, latero-uterine gestational sac with heart activity	Yes	Salpingostomy by laparoscopy	At day 8, a single continuing viable intrauterine pregnancy and a missed miscarriage of the second twin
Nguyen-Tran and Toy^18^	2000	?	?	?	Latero-uterine gestational sac with heart activity	?	Salpingectomy	She delivered healthy twins at term

All the cited literature are reports of unique cases.

The diagnosis should not be missed in cases of pregnant women with abdominal pain due to peritoneal irritation, even when they are referred with the diagnosis of possible appendicitis as in our case. The purpose of the treatment is to interrupt the development of the ectopic pregnancy and preserve the intrauterine pregnancy. Most cases of HP with tubal pregnancy have been treated surgically. The prognosis of the intrauterine gestation after treatment of the ectopic pregnancy is good. We observed high birth rates in this review above 90% (10/11).

## Conclusion

As a conclusion, it is important to emphasize the need for systematic exploration of the pelvis upon the first ultrasound scan of the pregnancy performed between 7 and 8 weeks of gestation, even if an intrauterine gestational sac is already confirmed, and even if there is no apparent risk factor. It is a fact that the diagnosis of HP tends to be overlooked after confirming the intrauterine pregnancy. When a diagnosis is established on time, the rate of pregnancies that reach term after treatment is significant.
